# Supplementary material for: A Global Screen for Assembly State Changes of the Mitotic Proteome by SEC-SWATH-MS
Source: Cell Syst. 2020 Feb 26;10(2):133–155.e6. doi: 10.1016/j.cels.2020.01.001 (PMC7042714; doi:10.1016/j.cels.2020.01.001)

P54252 | ATX3_HUMAN | ATXN3 ATX3 MJD MJD1 SCA3
Monomer MW [kDa]: 41.781 Monomer expected elution fraction: 48

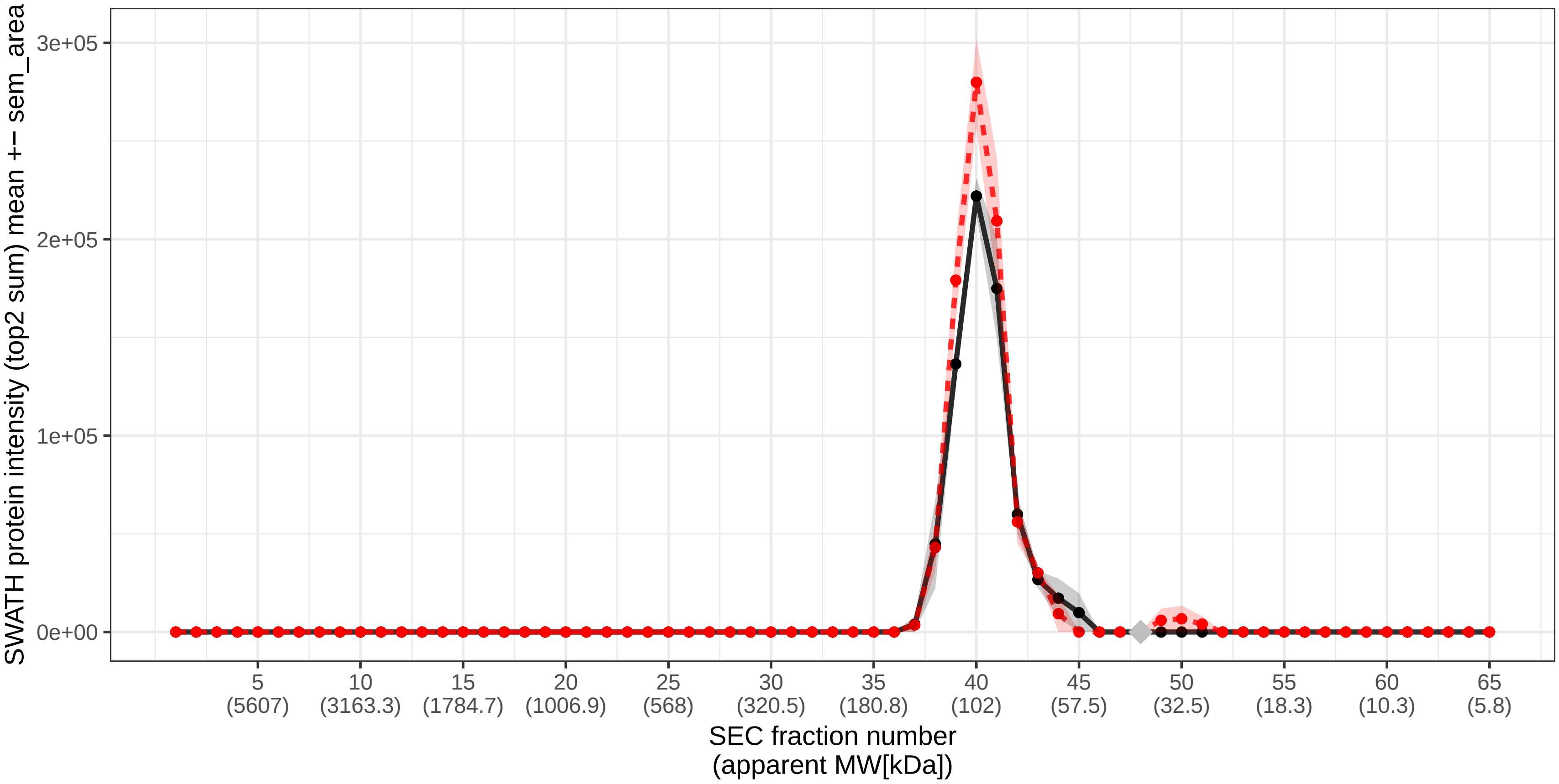

Supplement: Data S1. SEC-SWATH-MS Protein Chromatograms, Related to Figure 1 [file mmc6.zip › SECchrom_P54252_ATX3_HUMAN_ATXN3_ATX3_MJD_MJD1_SCA3.pdf]
